# Harvesting Effect and Extreme Temperature-Related Mortality in Italy

**DOI:** 10.1007/s10680-025-09764-4

**Published:** 2026-01-05

**Authors:** Vinod Joseph Kannankeril Joseph, Risto Conte Keivabu, Raya Muttarak, Emilio Zagheni, Stefano Mazzuco

**Affiliations:** 1 Department of Statistical Sciences “Paolo Fortunati”, University of Bologna, Bologna, Italy; 2 Department of Digital and Computational Demography, Max Planck Institute for Demographic Research, Rostock, Germany; 3 Department of Statistical Sciences, University of Padua, Padua, Italy

**Keywords:** Mortality, Extreme temperature, Harvesting effect, Italy

## Abstract

It is well-established that deaths peak in winter and show throughs in summer. However, it remains unclear how mortality patterns will unfold as the climate warms, bringing fewer cold days and more hot days. One concern is “harvesting,” where a short-term surge in deaths among the most vulnerable people is then followed by a period with fewer deaths than usual because those individuals would have died soon anyway. Under global warming, it is possible that higher mortality rates in summer will result not only from an increase in extreme heat events but also from a seasonal shift in excess deaths that would have previously occurred in winter. Combining mortality data from the Italian Statistical Office with temperature data from the Copernicus Data Store for Italy at the provincial level from 2011 to 2019, we employ Poisson regression models to estimate the effects of temperature extremes on mortality among individuals aged 60 and above. The results reveal that temperatures outside the comfort zone, both lower and higher, are associated with increased monthly mortality rates, with the strongest effects seen in the most extreme temperature ranges. We find evidence of a harvesting effect, particularly for moderately warm days (≥ 85th to < 95th percentile). However, even after high winter mortality, extremely hot days still lead to significant increases in deaths—especially among individuals aged 80 and above. This suggests that while some short-term mortality displacement occurs, it is not enough to offset the full impact of extreme heat, highlighting the continued vulnerability of older populations.

## Introduction

Temperature-related mortality, driven by both extreme cold and heat, has been extensively studied due to its significant public health implications (Atwoli et al., 2021; Basu & Samet, 2002; Gasparrini et al., 2015; Masselot et al., 2023; Son et al., 2019). In recent decades, heat-related mortality has risen globally, with a notable increase observed across all regions, including Italy (Ballester et al., 2023; García-León et al., 2024a; Lüthi et al., 2023; Vicedo-Cabrera et al., 2021). As climate change leads to fewer cold days, while intensifying extreme heat events, mortality patterns are expected to shift, with a higher concentration of deaths occurring in summer due to heat-related causes (Ha et al., 2011; Nordio et al., 2015; Zhao et al., 2021). This shift raises critical questions about the future of climate-related mortality. Will a decline in cold-related deaths offset, or even exceed, the rise in heat-related deaths?

In examining the future trajectory of climate-related mortality, the concept of the harvesting effect becomes particularly relevant. The “harvesting effect”, often called as “harvesting hypothesis” or “frailty effect”, refers to a temporary spike in mortality during or immediately after an extreme event, followed by a subsequent decline in deaths as individuals who were vulnerable due to underlying health conditions die earlier than expected (Luy et al., 2020; Qiao et al., 2015; Rocklöv et al., 2009; Stafoggia et al., 2006; Toulemon & Barbieri, 2008). The harvesting effect has gained renewed interest recently in the context of COVID-19 excess mortality assessments. In particular, researchers debated whether the pandemic would have led to a harvesting effect, where vulnerable individuals, who might have died later, succumbed earlier due to the virus (Cerqua et al., 2021; Lewnard et al., 2023; Riou et al., 2023). A similar question can be asked for temperature-related mortality: does an increase in deaths during winter or summer result in a temporary reduction in mortality afterwards? Despite its significance, this phenomenon remains understudied, particularly in relation to long-term climate trends. Italy, with an aging population and frequent extreme temperature events, is particularly vulnerable to temperature-related mortality (Ballester et al., 2023; EUROSTAT, 2023; García-León et al., 2024a), and an important case study, as it is a forerunner of demographic trends in other high-income countries.

This study addresses the gap in the literature by investigating the effects of temperature extremes affect mortality among individuals aged 60 and above, using granular mortality for 107 provinces of Italy over the period 2011–2019. Not only does Italy present substantial geographical variations characterised by three main climatic zones, but temperature-related excess mortality is also projected to be higher in Southern Europe as compared to other parts of the continent (Gasparrini et al., 2017). As cold spells are projected to persist under future warming (D’Errico et al., 2022), while the number of extreme hot days continue to increase, we examine the “harvesting effect” where temperature impacts on mortality differ depending on prior mortality levels. Specifically, summer temperatures influence mortality rates based on the mortality levels of the preceding winter, and winter temperatures affect mortality based on the mortality levels of the previous summer (Qiao et al., 2015; Rocklöv et al., 2009; Stafoggia et al., 2006; Toulemon & Barbieri, 2008).

Here, using data for the full Italian territory, we aim to test both how higher winter mortality affects the relationship between extreme temperature and mortality in the subsequent summer and likewise how higher summer mortality influences the temperature-mortality relationship in the subsequent winter.

## Background

### Temperature and Mortality

Extreme temperatures pose a significant threat to human health. The human body is equipped with several thermoregulatory mechanisms primarily governed by the hypothalamus to maintain a stable internal temperature despite exposure to varying external temperatures (Ahima, 2020). The key thermoregulatory functions include vasodilation and vasoconstriction, sweating, shivering and behavioural adjustments (Fischer et al., 2021). Prolonged exposure to extreme heat or cold impairs the body’s ability to regulate its internal temperature. It can increase the risk of heat-related illnesses such as heat exhaustion, heat stroke, and dehydration, as well as cold-related conditions like hypothermia and frostbite, and can become life-threatening (Barreca et al., 2016; Conlon et al., 2011; Fu et al., 2018; Khosla et al., 2021; Xia et al., 2023).

Historically, cold-related mortality has been more prevalent than heat-related mortality, accounting for a larger share of temperature-attributable deaths worldwide (García-León et al., 2024b; Zhao et al., 2021). Unlike heatwaves, which cause sudden spikes in mortality, cold exposure leads to a gradual increase in deaths (Alahmad et al., 2023). Only a small proportion of cold-related deaths result directly from hypothermia, while the majority are linked to cardiovascular or respiratory complications (Gasparrini et al., 2015). Cold temperatures constrict blood vessels, raise blood pressure, and increase the risk of heart attacks and strokes, particularly among older adults and individuals with pre-existing medical conditions (Arbuthnott et al., 2018). Additionally, respiratory infections, such as pneumonia and influenza, are more common in colder months, further increasing cold-related mortality (Masselot et al., 2025). At the same time, heatwaves trigger immediate spike in mortality due to heat exhaustion, heat stroke, and dehydration. Extreme heat also worsens cardiovascular diseases, raising mortality risks from ischemic heart disease and other related conditions (Dimitrova et al., 2021; Liu et al., 2022; Singh et al., 2024).

The relationship between temperature and mortality however is non-linear (Armstrong et al., 2014; Lüthi et al., 2023; Wang et al., 2017). Previous studies have found that this relationship often follows a U-shaped or J-shaped curve, depending on the climatic zone and/or sociodemographic subgroup studied (Armstrong, 2006; Barreca et al., 2016; Conte Keivabu, 2022; Hajat et al., 2014). That is, mortality rates tend to increase at both low and high temperature extremes, while they are generally lower at moderate temperatures. In other words, extreme heat and cold are associated with increased mortality risks (Curriero et al., 2002; Huynen et al., 2001; Martin et al., 2012). In Italy, this relationship follows a J-shaped curve, with a particularly heightened risk of mortality during extreme heat events (Michelozzi et al., 2006; Stafoggia et al., 2006).

In addition, the ability to adapt and cope with temperature extremes varies substantially with demographic and socio-demographic and economic characteristics (Muttarak et al., 2015), including factors such as age, sex, socio-economic status, geographic location, and pre-existing health conditions. Older people, pregnant women, young children, and those with pre-existing medical conditions are most vulnerable to heat stress (Dimitriadou et al., 2022; Hajat & Kosatky, 2010; Smoyer-Tomic & Rainham, 2001). For instance, with advancing age, older adults become more vulnerable to heat due to difficulty in thermoregulation and decreased sweating (Stafoggia et al., 2006). Likewise, most studies report higher female mortality during heatwaves due to physiological differences, higher prevalence of chronic diseases, socioeconomic vulnerabilities, and caregiving responsibilities (Achebak et al., 2020; Marí-Dell’Olmo et al., 2019; van Steen et al., 2019; Vésier & Urban, 2023). However, some studies suggest that in regions with improved public health systems, resilient infrastructure, effective emergency responses, and adaptive measures, temperature extremes may not significantly increase mortality rates (Barnett et al., 2010; Gasparrini et al., 2015; Kinney et al., 2008; Vicedo-Cabrera et al., 2018). Given this complexity, statistical models that assume non-linearity are needed to flexibly characterize temperature-mortality association (Curriero et al., 2002; Kunst et al., 1993). These examples highlight the importance of considering demographic heterogeneity in studying temperature-related mortality.

### Seasonal Patterns of Mortality

Mortality patterns are not constant and fluctuate throughout the year, with temperature-related deaths varying significantly from one season to another. Several studies have shown seasonal patterns of mortality, with higher mortality rates in winter and lower in summer (Gemmell et al., 2000; Hajat & Kosatky, 2010; Momiyama & Katayama, 1972; Rau et al., 2018). Studying these seasonal mortality patterns helps identify and quantify how short-term mortality spikes influence long-term mortality trends and effect modification—that is, whether the strength of the temperature–mortality relationship changes depending on mortality levels in the preceding season (Ha et al., 2011; Rocklöv et al., 2009; Stafoggia et al., 2009).

The excess winter deaths in Europe are typically attributable to the peak in influenza and other infections, which significantly contribute to cold-related mortality rather than to the low temperatures of the season (Michelozzi, 2016; Nielsen et al., 2019; Vestergaard et al., 2017; Walkowiak et al., 2024). Also, the mortality rates vary considerably between summer and winter, with differences observed between northern and southern countries. In the north, mortality rates typically increase during the winter due to extreme cold weather. Research indicates that the cold-related mortality exceeds heat-related mortality in northern European countries (Keatinge et al., 2000; Masselot et al., 2023). The south, with its milder winters, experiences less of an increase in winter mortality, but shows higher mortality rates due to heat-related illnesses in summer. (Dimitriadou et al., 2022; Fowler et al., 2015; Madaniyazi et al., 2024; Walkowiak et al., 2024).

Apart from variations by geographical locations, the timing of extreme temperature exposure during the year also influences mortality risks. Heat waves in spring or early summer, for example, often result in more deaths than those occurring later in the summer (Wolfe et al., 2001). People who survive the heatwaves in later summer may have developed coping mechanisms or physiological adaptations following initial exposure to high temperatures (Basu & Samet, 2002). It is also possible that the most vulnerable individuals are more affected by earlier heat waves, leaving fewer susceptible persons alive later in the season.

### Harvesting Effect

A handful of studies have estimated that exposure to high temperatures and extreme weather events is associated with increased mortality in the near term (Anderson & Bell, 2011; Arsad et al., 2022; Ballester et al., 2023; Gasparrini et al., 2015; Michelozzi, 2004; Toulemon & Barbieri, 2008). In many cases, this increase can be counteracted by a much lower mortality rate than expected in the days and weeks following the exposure. This is likely due to the lower stock of frail individuals due to their early death in a previous shock (Luy et al., 2020; Qiao et al., 2015; Rocklöv et al., 2009; Stafoggia et al., 2009; Toulemon & Barbieri, 2008). To date, there is limited evidence on this phenomenon where higher levels of deaths in a specific time of the year determines lower mortality in subsequent months of the same year due to the decline in the stock of individuals who are particularly vulnerable and severely ill.

Understanding the harvesting effect is essential for defining assumptions for population projections under climate change (Muttarak, 2021). As growing evidence suggests that heat-related mortality is likely to rise in the future, it is important to consider the potential role of the harvesting effect, that is, whether and how shifts in seasonal conditions may influence mortality patterns. For example, milder winters might allow frail individuals to survive longer, potentially increasing their likelihood of succumbing to summer heatwaves. Conversely, more frequent and intense heatwaves induced by climate change could lead to higher mortality during the summer months, thereby reducing the pool of vulnerable individuals exposed to winter cold. In either case, estimates of age- and sex-specific mortality rates in a given calendar year may remain relatively stable, as excess mortality simply shifts from one season to another. However, understanding who is vulnerable (and when) is key to make sound population projections, especially over the longer term and in light of demographic shifts. Studying the harvesting effect is therefore critical as the lack of evidence males it difficult to form realistic assumptions about how mortality patterns may evolve under future climate change.

While numerous studies have identified seasonal patterns of mortality, only a few of them have examined how higher mortality rate in winter might influence mortality rates in summer, and vice versa. Existing studies mainly investigate the harvesting effect caused by specific events such as seasonal influenza (Lytras et al., 2019), COVID-19 (Cerqua et al., 2021; Rivera et al., 2020), and air pollution (Schwartz, 2000; Zeger et al., 1999), heat waves or cold spells (Baccini et al., 2013; Cheng et al., 2018; Grize et al., 2005; Qiao et al., 2015; Stafoggia et al., 2009; Toulemon & Barbieri, 2008). The findings on the harvesting effect are mixed, with some studies reporting a strong evidence, while others suggest a more limited impact (Rocklöv et al., 2009; Stafoggia et al., 2009; Toulemon & Barbieri, 2008). A limited number of studies focusing on seasonal patterns have reported evidence of the harvesting effect. Ha et al. (2011) found in their study in Korea that the risk of heat-related deaths in summer was higher after a winter with low mortality, compared to one with high mortality. This suggests that low winter mortality leaves more individuals susceptible to heat in the following summer, while high winter mortality reduces this risk. Similarly, a study conducted in Stockholm, Sweden found that the severity of temperature on mortality in summer depends significantly on the extent of respiratory, cardiovascular, and influenza mortality during the previous winter (Rocklöv et al., 2009). Similarly, Qiao et al. (2015a) reported that in Brisbane, Australia (1996–2004), heat-related mortality was more pronounced in summers following winters with lower mortality rates.

On the other hand, in their study on the mortality impact of the August 2003 heat wave in France, Toulemon and Barbieri (2008) observed that the harvesting effect was modest. They suggested that, although there was an immediate spike in mortality following the heatwave, it may have been offset by delayed deaths, as some individuals became frail due to the heatwave and died in its aftermath. To our knowledge, the only study on Italy examines Rome between 1987 and 2005, finding that higher winter mortality is associated with a weaker relationship between heat and mortality in the following summer (Stafoggia et al., 2009). Our study addresses this gap by extending the analysis to the entire country using granular mortality data covering the period between 2011 and 2019.

### Italian Context

**Fig. 1:**
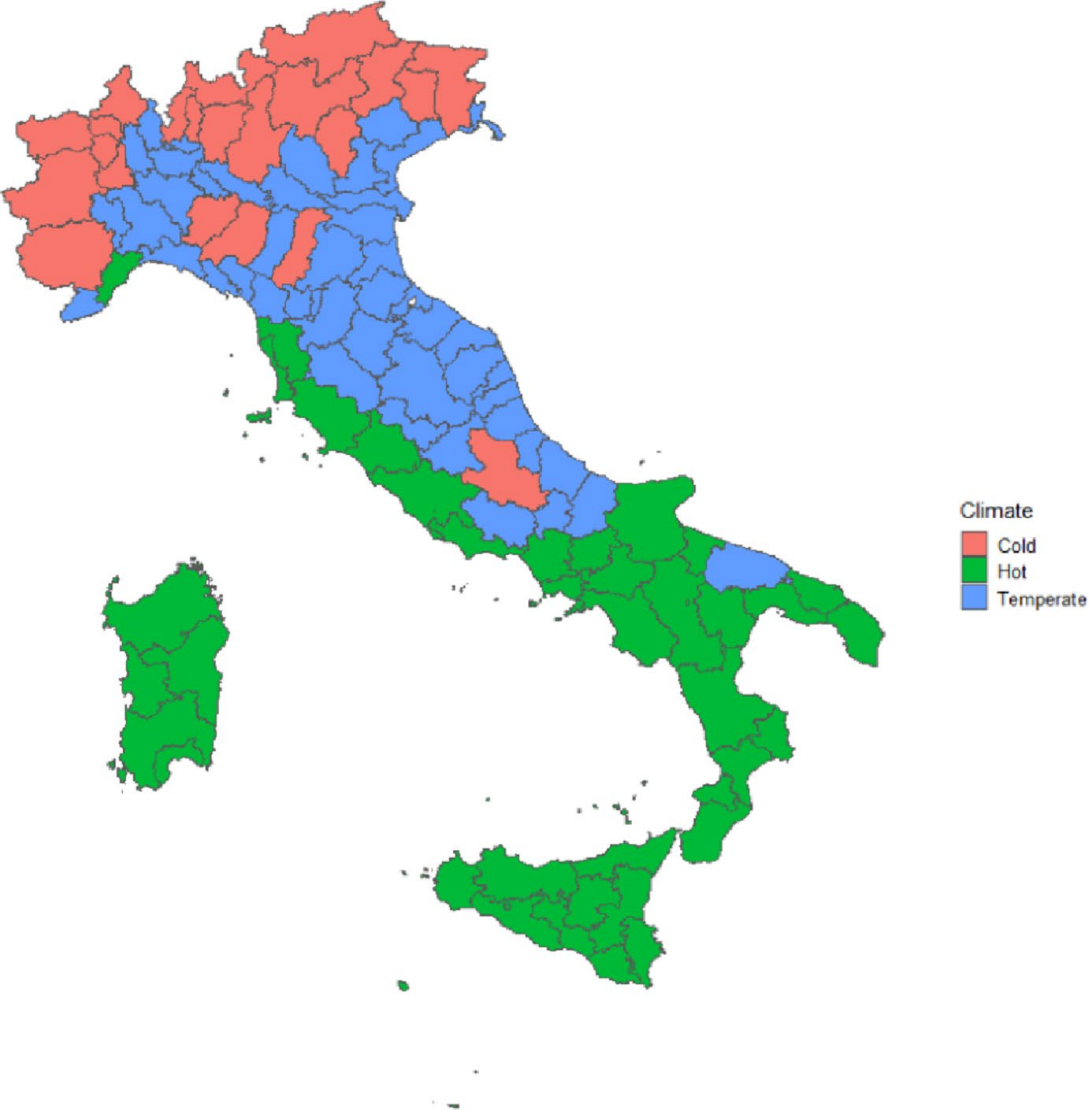
Climatic regions in Italy. The figure shows the Köppen-Geiger climate classification based on Beck et al. (2018), grouping provinces into three climate zones: hot, characterized by hot summers and mild winters (Bwh, Bwk, Bsh, Bsk, Csa, Csb); temperate, with moderate temperatures and no extreme seasonal variation (Cfa, Cfb, Cfc); and cold, marked by long, cold winters and shorter cool summers (Dsa, Dsb, Dsc, Dfa, Dfb, Dfc, eT, eF).

Italy lies geographically in a temperate zone and is known for its Mediterranean climate, especially along the coasts. In Fig. 1, we depict the geographical distribution of climatic zones in Italy across the 107 Italian provinces. We observe colder regions in the northern mountainous parts. Temperate climates are located in the central eastern parts of the country whereas the south and centre-west host the hottest climatic conditions. Besides having climatic differences, Italy is a country that has well-documented regional differences in terms of demographic patterns, well-being, economic performance, and institutional strength (Fina, 2021).

The adverse effect of high temperatures on mortality in Italy is well documented with studies highlighting both cold-related and heat-related deaths. A study by de’Donato et al. (2013) documented increased mortality and emergency visits among the elderly during a cold spell in February 2012, across 34 Italian cities. In contrast, heat-related mortality has also become increasingly prominent, especially during extreme heat events. A study on seasonal mortality across 32 Italian cities found an 11% excess mortality in 2015 due to seasonal temperature variations, with summer deaths rising by 10% primarily from heat waves (Michelozzi, 2016). The summer of 2003 was particularly devastating, with a significant relationship between high temperatures and increased mortality in Rome, primarily affecting the older adults and those of lower socio-economic status (Michelozzi, 2004).

A number of studies have further shown the impact of heat on population subgroups. Ishigami et al. (2008) identified age as a significant risk factor for heat-related deaths in Milan, noting that socio-economic status modifies this effect. Stafoggia et al. (2006) observed varying effects of summertime high temperatures on daily mortality across different demographic and health subgroups in four Italian cities, particularly affecting elderly individuals, women, widows, widowers, and those with psychiatric or cardiovascular conditions. Ballester et al. (2023), in their recent study to quantify heat-related mortality burden during the summer of 2022 in Europe, found that the highest summer heat-related mortality rates were in countries near the Mediterranean Sea. Given variations in climatic zones in Italy, we may observe differentials in cold- and heat-related mortality across regions. Italy had the highest number of heat-related deaths among the 65+ population in all of Europe. These deaths can not only be attributed to the climatic vulnerability and high frequency and intensity of extreme weather events in Italy, but also to the fact that Italy is one of the world’s fastest-aging countries, with a median age above 48 years, and 24% of residents older than 65 (EUROSTAT, 2023). Older individuals are consistently identified as the most vulnerable to temperature extremes (Stafoggia et al., 2006). With its relatively high proportion of older adults, Italy hence stands out as one of the most susceptible countries in Europe to temperature-related health impacts.

## Data, Variables and Methods

We analyse the impact of temperature extremes on mortality among Italians aged 60 and above from 2011 to 2019 using mortality data from ISTAT and temperature data from E-OBS at the provincial level. We employ Poisson regression models with fixed effects to estimate the effect of temperature on mortality, incorporating temperature bins, sociodemographic factors, and environmental variables, with province-by-month and month-by-year fixed effects. To examine the effect of previous winter mortality on the temperature-mortality relationship in the following summer, an interaction term between temperature bins and a high winter mortality indicator is added in the model.

### Data

We use Italian register data on all-cause mortality provided by ISTAT – Italian National Institute of Statistics (Istituto Nazionale di Statistica). In our analysis, we focus on deaths of individuals aged above 60, from 1st December 2011 to 31st August 2019 localized at the provincial level. There are 107 provinces in Italy in total. The period chosen for the analysis includes eight full winters necessary for our analysis of the harvesting effect. The data is divided into four age categories (60–69; 70–79; 80–89; 90+) and by two sex^1^ categories. Also, we collect from ISTAT yearly statistics on the population residing in the provinces by the corresponding age categories and sex.

The meteorological data used is provided by the E-OBS (Cornes et al., 2018) and available in the Copernicus Data Store. This data is gridded and based on interpolated data retrieved from a large array of weather stations distributed within the European territory. The spatial resolution of the data is of about 11 km, and it comprises complete and reliable daily data from 1950 on a large number of meteorological variables. For the purpose of our study, we collect data from 1st December 2011 to 31st August 2019 on mean daily temperature, precipitation, humidity, solar radiation and wind speed. We allocated the values of the gridded meteorological data to the 107 Italian provinces calculating the average values of the grids falling within the administrative boundary of each province.

Air pollution is found to be one important factor influencing the association between temperature and mortality (Zhang et al., 2024). Studies have documented the correlations between rising temperatures and increased mortality due to air pollution (Liu et al., 2022; Stafoggia et al., 2023; Zhang et al., 2024). Air pollution can exacerbate the adverse health effects of extreme heat, resulting in higher mortality rates among vulnerable populations and those with pre-existing cardiovascular or respiratory conditions (Bell et al., 2013). To account for this, we collected monthly average data on Particulate Matter 2.5 (PM2.5), one of the most harmful pollutants to human health (Fang et al., 2016, p. 5). The PM2.5 data for Italy was sourced from the CAMS global reanalysis (EAC4) monthly averaged fields, provided by the Copernicus Atmosphere Data Store. The data are gridded with a spatial resolution of approximately 75 km x 75 km and are available on a monthly basis from 2003. For our analysis, we used data from December 2011 to August 2019, averaging the values of the grids corresponding to each province.

#### Variables

Our main outcome of interest is the monthly death rate by age and sex in each province. We aggregated daily deaths for the population aged 60 and above into four age categories (60–69; 70–79; 80–89; 90+) by sex over 93 months (December 2011 to August 2019) for 107 provinces comprising a total of 79,608 observations. To compute population exposures, annual population figures were divided by twelve to obtain monthly estimates. The values between years for each demographic group and province were interpolated.

Daily mean temperatures recorded in each province are used to construct monthly temperature bins based on percentiles of the province-specific temperature distribution during the study period. Specifically, nine temperature bins were created. They include, respectively, the number of days in which the provincial temperature falls below and equals the 5th percentile, from the 5th to the 10th percentile, from the 10th to the 15th percentile, from the 15th to the 20th percentile, above the 20th but below the 80th percentile, from the 80th to the 85th percentile, from the 85th to the 90th percentile, from the 90th to the 95th percentile, and above the 95th percentile. Days with temperatures between the 20th and 80th percentiles are considered the comfort zone and set as the reference category. Using temperature bins allows us to capture the non-linear relationships between temperature and mortality as previously described in previous studies (Conte Keivabu, 2022). Also, using percentiles from the temperature distribution within each province enables us to capture location-specific extreme temperatures, accounting for variations in temperature-mortality response functions across provinces that might vary due to the differences in the adaptation to the local temperature (Masiero et al., 2022). The aim of this study is to capture locally extreme temperatures relative to each province’s recent climate, reflecting differences in adaptation across Italy. This approach is standard in temperature and mortality research and avoids mixing long-term climate shifts with short-term variability (Conte Keivabu, 2022; Conte Keivabu et al., 2024). Using long-term term trends could classify many recent warm days as extreme even if people have already adapted.

Additionally, we include control variables for several meteorological variables. We capture average monthly precipitation, solar radiation, humidity, wind speed and air pollution PM2.5 following the specification strategy of Conte Keivabu (2022). To study the harvesting effect, we assess whether higher mortality in the previous winter affects the temperature mortality association in the following summer. To do so we ranked 8 winters based on their mortality rate. More precisely, we use a meteorological definition of winters spanning the months of December to February. In our data, the first winter is that of December 2011 to February 2012 and the last is the winter spanning from December 2018 to February 2019. We ranked each province’s eight winters based on their mortality rate and classified them into two groups “High Winter Mortality” when ranked first to fourth and “Low Winter Mortality” when ranked fifth to eighth. Consequently, we observe how winter mortality modifies the temperature-related mortality in the following summer. In other words, if there is a harvesting effect, we should observe a weaker relationship between temperature and mortality in the summer following a particularly high winter mortality and vice versa with low winter mortality (Stafoggia et al., 2009).

### Empirical Strategy

Poisson regression models with fixed effects are employed to estimate the effect of temperature on mortality. It is a standard approach for modelling mortality counts, and using a population offset makes it mathematically equivalent to modelling mortality rates(Conte Keivabu, 2022; Conte Keivabu et al., 2024). This method is appropriate for count data and allows us to control for unobserved seasonal and provincial differences that could bias the temperature and mortality association. The equation can be described as follows.

1 $$\:Log\:\left({Y}_{ptas}\right)=Log\:\left({E}_{ptas}\right)+\sum\:_{j}{\theta\:}_{j}{TEMP}_{pt}^{j}\:+\:{\beta\:X}_{pt}+\:{\alpha\:}_{pm}+\:{\delta\:}_{ym}$$

$\:{Y}_{ptas}$ denotes the count of deaths in province $\:p$, time *t*, age $\:a$ and sex $\:s$. $\:{E}_{ptas}$ is introduced as an offset term capturing the population exposed to the risk of death in each province, time and demographic group. $\:{TEMP}_{pt}^{j}$ represents the monthly temperature bins of the province specific percentiles in the temperature distribution We include a vector of control variable $\:{\beta\:X}_{pt}$ representing the demographic categories for age and sex, and the meteorological variables to capture biases due to other possible confounding factors. Furthermore, we introduced $\:{\alpha\:}_{pm}$ to capture province-by-month fixed effects and $\:{\delta\:}_{ym}$ to capture month-by-year fixed effects, and cluster standard errors at the province level to correct for autocorrelation within province over time. The fixed effects are used to control for potential time-varying factors and seasonal trends that may be correlated with temperature and mortality across all provinces observed over time.

To test the modifying effect of previous winter mortality on the temperature-mortality relationship in the following summer we estimate Eq. (2):

2 $$\:Log\:\left({Y}_{ptas}\right)=Log\:\left({E}_{ptas}\right)+\sum\:_{j}{\theta\:}_{j}{TEMP}_{pt}^{j} \times {WinterMort}_{pt}+\:{\beta\:X}_{pt}+\:{\alpha\:}_{pm}+\:{\delta\:}_{ym}$$

where we run a model similar to the one described in Eq. (1), but only for the summer months adding an interaction between the temperature bins and a binary indicator describing the previous winter as having high winter mortality (Yes = 1) in province *p* and time *t*. As we focus on the summer months, we capture exposure only to temperature from the 80th to the 85th percentile, from the 85th to the 90th percentile, from the 90th to the 95th percentile, and above the 95th percentile.

## Results

In Table 1 we present the summary statistics of monthly provincial averages for the outcome and explanatory variables. As summarized in Table 1, the number of days assigned to each temperature bin shows marked variation across provinces and years, highlighting substantial heterogeneity in exposure to different temperature conditions. Similarly, we observe large variation in death counts. For instance, the highest number of deaths, 1,091, was recorded in Rome among women aged 80–89, in January 2017. We depict the trend in mortality in Rome across the study period in Fig. 6 for all deaths and observe the highest number of deaths in this month. Conversely, the lower population exposure, 21, was observed in Valle D’Aosta in the age category 90 + for men in December 2011. Additionally, we provide the monthly provincial averages or meteorological variables like precipitation, solar radiation, humidity, wind speed and air pollution PM2.5.

**Table 1:** Summary statistics of the monthly provincial averages for the main variables.

	Mean	SD	Min	Max
Death counts	57.1	69.96	0.00	1,091
Population	1,646	2,199	21	22,129
< 5th percentile	1.64	3.86	0.00	26
5th to 10th percentile	1.52	2.78	0.00	16
10th to 15th percentile	1.51	2.46	0.00	15
15th to 20th percentile	1.49	2.39	0.00	16
80th to 85th percentile	0	0	0.00	0
85th to 90th percentile	1.52	2.61	0.00	16
90th to 95th percentile	1.53	2.83	0.00	18
>95th percentile	1.59	3.2	0.00	18
Solar radiation	16.4	7.84	3.49	33.02
Precipitation	0.25	0.21	0.00	2.22
Wind speed	2.44	0.63	0.77	7.11
Humidity	72.7	7.83	43.22	92.73
PM2.5	16.02	5.38	4.23	41.38
Number of month-years	93			
Sex categories	2			
Age categories	4			
Number of provinces	107			
Total observation	79,608			

**Fig. 2:**
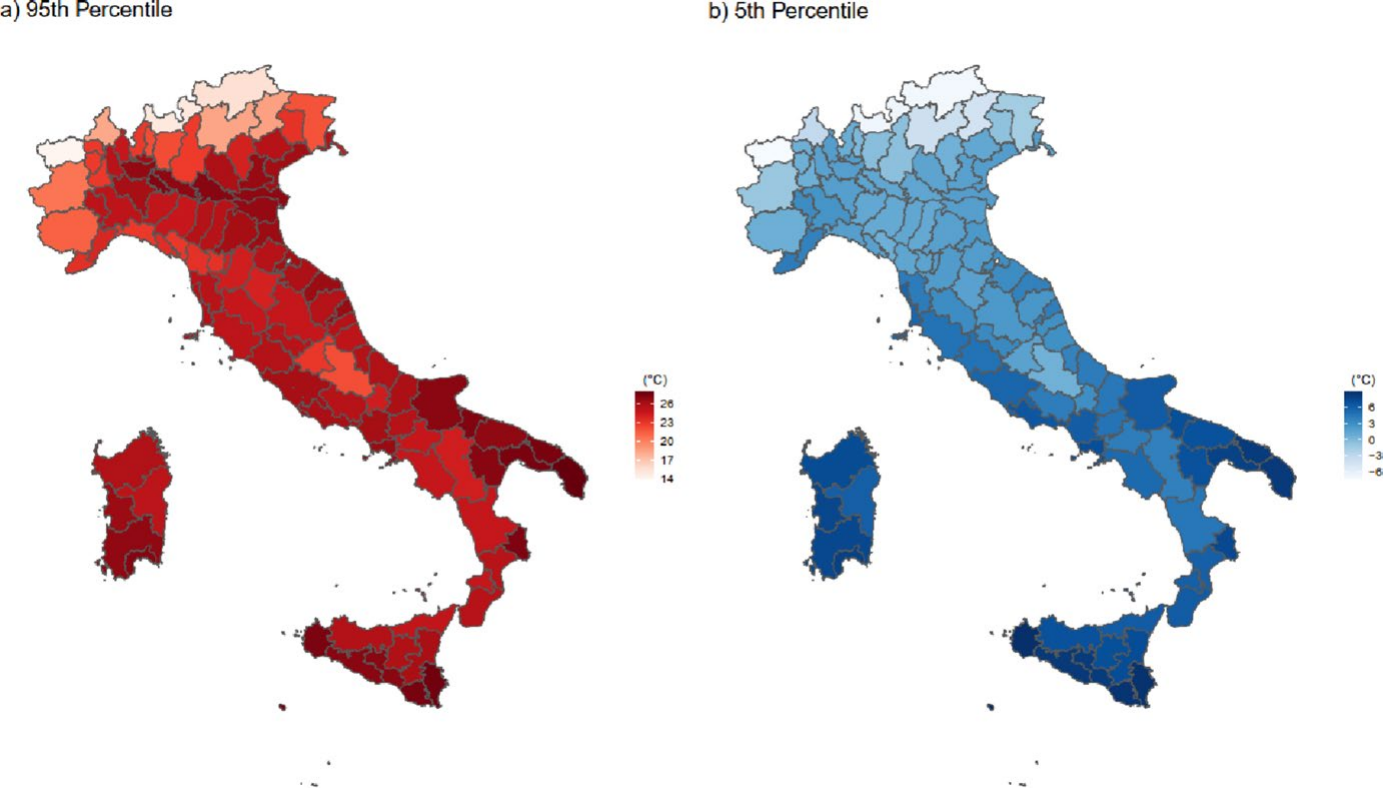
Map of 5th and 95th percentiles of mean temperature in Italian provinces. 95th and 5th percentiles in the mean temperature distribution in provinces 2011 to 2022.

Figure 2 and Table 11 presents the 95th and 5th percentiles of mean temperatures across Italy. The maps reveal significant variations in temperature distribution across 107 provinces. In the hottest 95th percentile, mean temperatures reach nearly 30 degrees Celsius in provinces such as Lecce, Taranto, Brindisi in the region of Puglia, or Siracusa and Ragusa in Sicily. At the same time, in the coldest 5th percentile, temperatures drop as low as − 6 °C in alpine provinces like Aosta, Sondrio, and Bolzano.

**Fig. 3:**
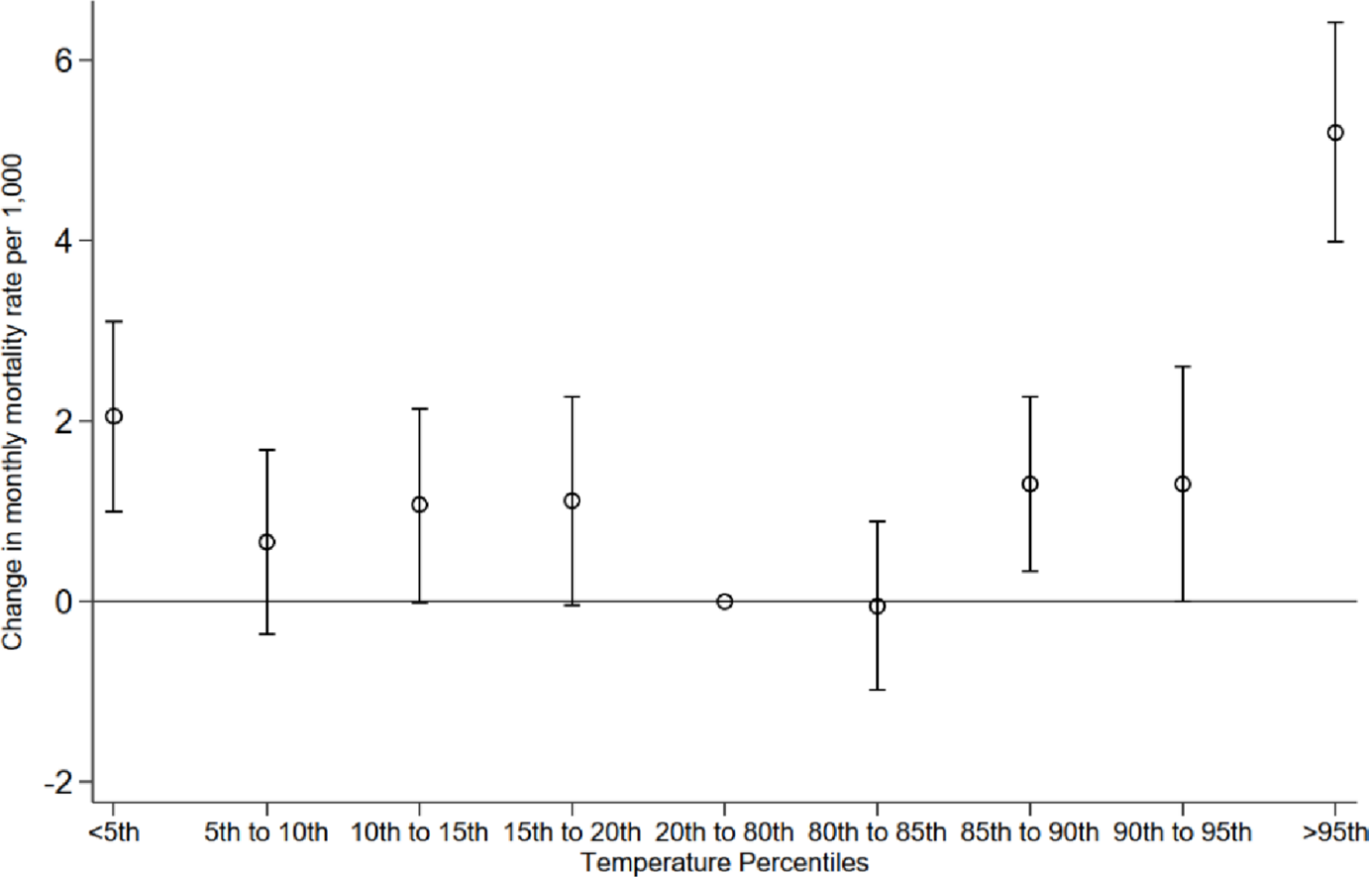
Extreme temperature and mortality rates. The results were obtained by Eq. (1) and plotting the coefficients for temperature bins with 95% confidence intervals.

Figure 3 presents a plot of coefficients obtained from the regression analysis of mortality and temperature variation for the pooled sample (see Appendix Table 2 for the full results). Temperatures preceding and exceeding the comfort zone are associated with an increased mortality rate, with a more robust and pronounced effect size observed in the most extreme temperature bins. Specifically, for cold days we observe a substantive effect size below the 5th percentile, representing an increase in the monthly mortality rate of approximately 2 per 1,000. And for hot days, when temperatures are at or above the 95th percentile, this corresponds to an increase in the monthly mortality rate of approximately 5 per 1,000.

**Fig. 4:**
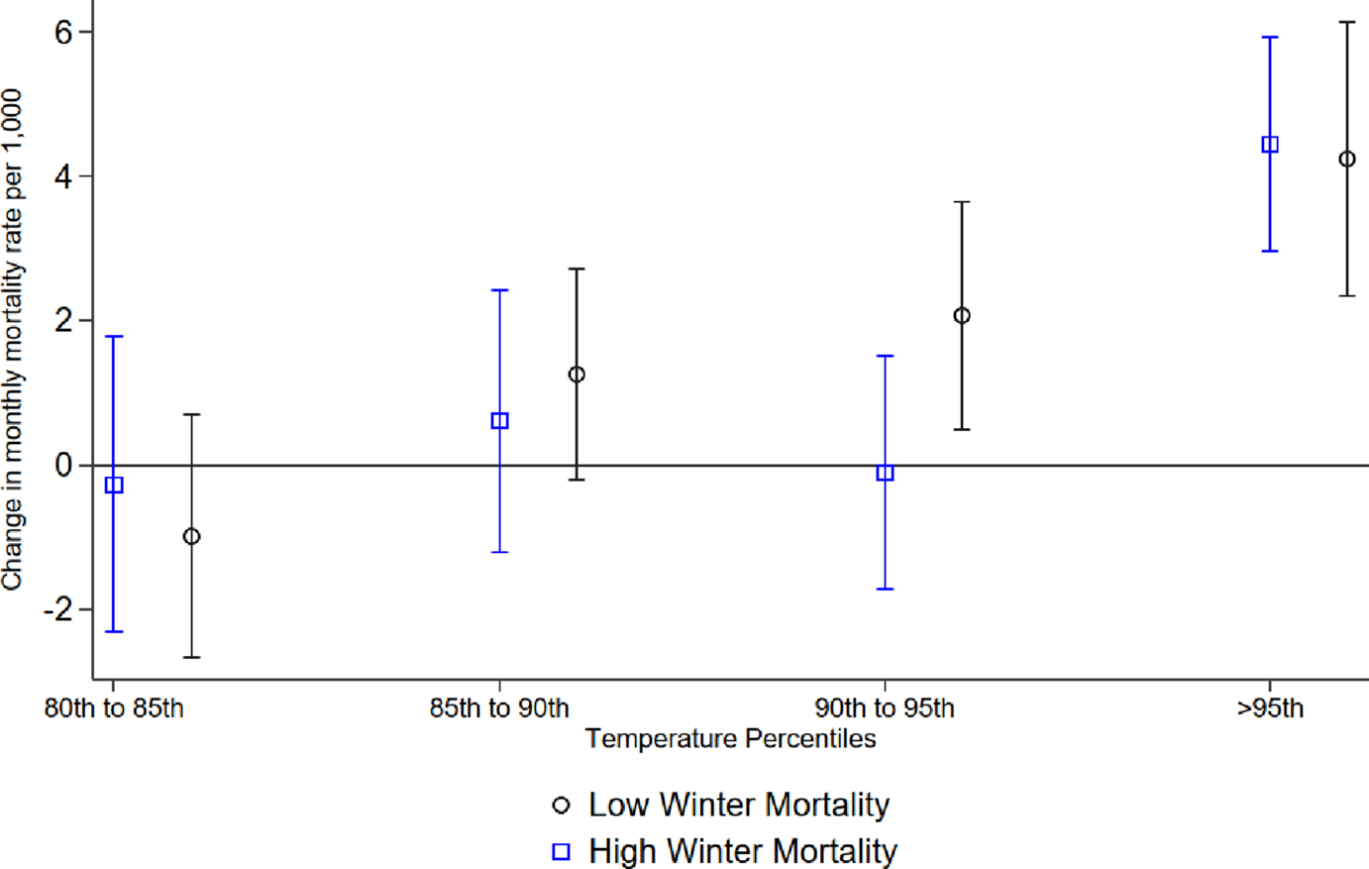
Heat and mortality in summer by winter mortality. The analysis is based on Eq. (2) for the summer months. Low winter mortality refers to the four winters with the lowest mortality, and high winter mortality refers to the four winters with the highest mortality. The coefficients estimated for each temperature bin are plotted together with 95% confidence intervals.

Figure 4 (Appendix Table 3) presents the results of the interaction between temperature bins and mortality during the summer season along with interaction with the previous winter mortality. The results suggest warm days (≥ 90th and < 95th ) impact mortality only following winters with low mortality. Conversely, we observe similar impacts of hot days (> 95th ) on mortality regardless of the mortality rate of the previous winter. This pattern suggests the presence of a harvesting effect, where frail individuals who survived winter are susceptible to the impacts of warm days (> 90th and 95th ) in the subsequent summer. Consequently, when a winter has higher-than-expected mortality, the population pool of susceptible individuals is smaller, leading to lower mortality rates during subsequent seasons with exposure to warm days (> 90th and 95th ), suggesting a lower threshold of resistance to heat in this population. In contrast to moderately warm days, the effect of extremely hot days (> 95th percentile) remains strong regardless of winter mortality. This indicates that even after high winter mortality, extreme heat continues to cause substantial increases in deaths.

**Fig. 5:**
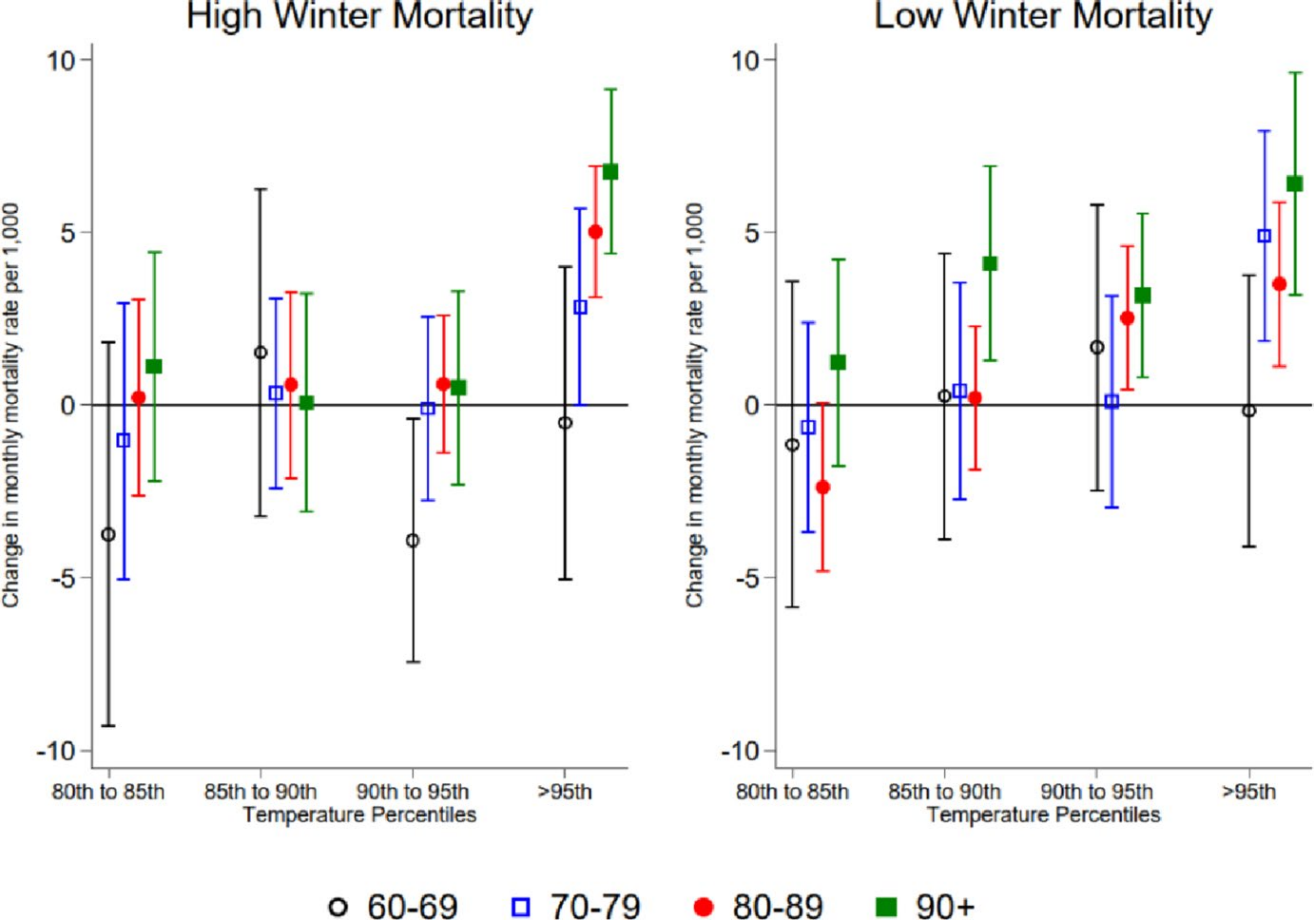
Heat and mortality in summer by winter mortality and age categories. The results were obtained by Eq. (2) for the summer months and separately by age groups (60–69; 70–79; 80–89; 90+). Respectively, the low winter mortality refers to the four winters with the lowest mortality, and High Winter mortality to four years with highest levels of winter mortality. We plot the coefficients for temperature bins with 95% confidence intervals.

**Fig. 6:**
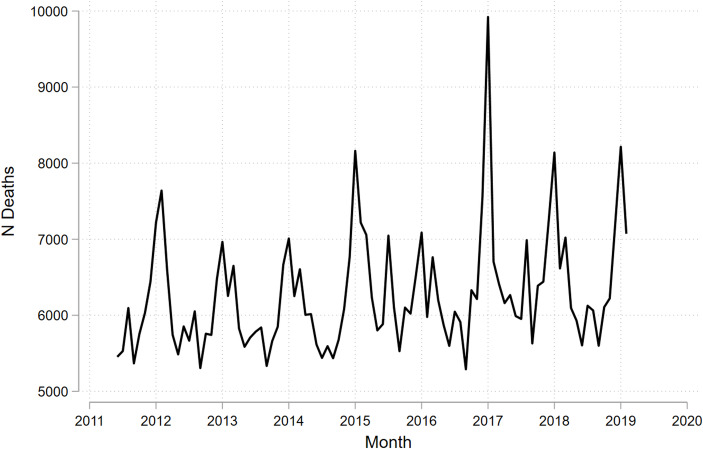
Monthly number of deaths in the province of Rome age 60+. In the figure we provide the total number of deaths for individuals aged 60 and above in the province of Rome from 1st December 2011 to 31st August 2019.

Figure 5 (Appendix Table 4) presents results of the interaction between temperature bins and mortality during the summer season along with interaction with the previous high mortality winters run separately by age groups (60–69; 70–79; 80–89; 90+). Notably, with low winter mortality, there is a consistent increase in mortality rates with higher temperature percentiles, especially in older age groups (80–89 and 90+). Nevertheless, the group difference is statistically significant only when we look at the exposure to temperatures in the 80th to 85th percentile bin and the age group above 90 with higher winter mortality.

## Robustness Checks

The effect modification of temperature on mortality due to previous winter mortality could be expected also due to variations in summer mortality. As done for winter mortality, we ranked summers (June to August) starting from 2011 to 2018, based on their mortality rates. We created in this way a binary indicator for High and Low Summer Mortality and replicated the results described in Eq. (2) but with temperatures in winter. In Appendix Table 5, we present the results of the association between temperature bins and mortality during the winter season along with an interaction with the previous summer mortality. We do not observe that summer mortality substantively modifies the association between temperature and mortality in winter.

As observed with temperature, we could expect lower winter mortality to modify the impact of air pollution on mortality in the following summer as well. We test for an effect modification of previous winter mortality on the impact of PM2.5 on mortality adding an interaction with our binary indicator of winter mortality with PM2.5. In Appendix Table 6, we observe a similar pattern observed with heat. More precisely, higher mortality in winter appears to reduce the increase in mortality due to PM2.5. These findings support the concept of harvesting effect, where an increase in deaths in winter among frail individuals is followed by a subsequent decrease in expected deaths in the following summer (O’Neill et al., 2003; Schwartz, 2000; Zeger et al., 1999).

Additionally, we explore heterogeneous impacts of extreme temperatures on mortality by age and sex. In Appendix Table 7, we present the results of the interaction between temperature bins with age. The coefficients indicate how the effect of extreme temperatures on mortality rates differs across different age groups. The results suggest that individuals aged 80–89 and 90+ have higher mortality rates related to both extreme cold and hot days. In Appendix Table 8, we present the results of the interaction between temperature bins and sex. The results show a stronger effect of cold and especially heat for women. The results for hot days are consistent with results of previous studies reporting a larger burden of heat for women in Italy (de’Donato et al., 2013; Ishigami et al., 2008). To summarize both extreme cold and extreme heat are associated with increased mortality. This effect is more pronounced for females across several temperature ranges, particularly at the lowest and highest extremes.

Italy hosts a varied spectrum of climatic areas as we showed in Fig. 1, that could determine differences in the relationships between temperature and mortality. We investigate this interacting the temperature exposure with the three climatic areas. In the Appendix Table 9, we observe a heterogenous impact of cold temperatures on mortality, but not major differences in the effect of hot temperatures by climatic zones. More precisely, days below the 5th percentile show to increase mortality in the Temperate and Hot climatic zones, but not in the Cold zone. These findings are in line with previous studies on climate adaptation and suggest lower adaptation to cold days in the hotter areas of the country that are less used to colder weather. Additionally, we analyse the harvesting effect separately by climatic zones. In Appendix Table 10, we do not observe any major differences in the harvesting effect by climatic zones.

## Discussion and Conclusions

We first examined the effect of exposure to monthly temperature variations on mortality of the Italian population aged 60 years and older. The results revealed a J-shaped relationship between temperature and mortality in Italy, indicating a higher risk of death associated with extreme heat. These findings are consistent with previous studies that have reported similar patterns in temperature-related mortality in Italy (Michelozzi et al., 2006; Stafoggia et al., 2006). Overall, these results provide insights into how the relationship between extreme temperatures and mortality rates may vary across different age groups and sex, allowing for a more nuanced understanding of the impact of temperature extremes on different demographic groups (Dimitriadou et al., 2022; Hajat & Kosatky, 2010; Muttarak et al., 2015). These results are vital for understanding the immediate impact on mortality rates and informing future public health policies and interventions (Fox et al., 2019). Extreme temperatures have a negative impact on health and mortality, and the associated health burden is expected to increase with climate change, especially under the most extreme scenarios of global warming (Basu & Samet, 2002; European Environment Agency, 2023; Romanello et al., 2022).

The aim of this study was to assess the harvesting effect, expecting an effect modification of previous winter mortality on the association between temperature and mortality in the following summer. Then we examined the hypothesis that the impact of summer temperature on mortality varies based on the mortality level observed in the preceding winter. Our findings show that the effects of warm temperatures are more pronounced after winters with low mortality, especially among older age groups. This may be because high winter mortality reduces the pool of vulnerable individuals, thereby diminishing the impact of high temperatures on mortality in the following summer. These findings are consistent with the earlier studies conducted in Korea and Rome in Italy (Ha et al., 2011; Stafoggia et al., 2009). The strong seasonality in mortality patterns across different subgroups can be partly explained by a mortality displacement mechanism (Qiao et al., 2015; Rocklöv et al., 2009). Furthermore, the pool of susceptible and vulnerable populations changes over time in response to the intensity and severity of consecutive extreme events (Chambers, 2020).

Although there is a significant effect of winter harvesting in reducing mortality rates in summer, people still succumb to extreme heat. This can be explained by the frailty hypothesis in demographic and epidemiological research, which suggests that when an extreme event occurs (such as a heatwave or a severe cold spell), it disproportionately affects vulnerable populations, including those with pre-existing health conditions, advanced age, or genetic predispositions, making them more “frail” (Heuberger, 2011; Russo & Bisanti, 2004; Zhou et al., 2023). As a result, even though the pool of susceptible and vulnerable populations declines due to high mortality during severe cold spells, the pool of healthy individuals becoming frail soon after the cold spell increases (Dent et al., 2020; Nakajima et al., 2018; Willand et al., 2017). Due to their persisting high frailty condition, their deaths are accelerated when they are exposed to extreme heat in the following season (Hajat et al., 2005). Also, there can be the influence of “residual frailty”. This is referred to as the state where individuals have been weakened by an external stressor but have survived, potentially making them more susceptible to future adverse events (Qiao et al., 2015; Walkowiak et al., 2024).

Although we observe a significant winter harvesting effect that reduces summer mortality, suggesting short-term mortality displacement after cold seasons, this pattern does not hold in the opposite direction. High mortality during hot summers does not lead to a decline in winter deaths, especially on extremely cold days. This indicates that vulnerable individuals continue to succumb to cold, even if a portion of the frail population may have already died during extreme summer. This can because exposure to extreme events disproportionately affect frail individuals (e.g., the elderly, those with chronic conditions), the populations vulnerable to cold and heat may not completely overlap. For example, individuals who survive a hot summer particularly older adults or economically disadvantaged people may still face substantial risks during winter due to factors like inadequate access to heating or insulation. Moreover, the causes of death also vary by season, with summer mortality is often linked to cardiovascular stress and dehydration, and cold to respiratory illnesses like pneumonia and influenza (Masselot et al., 2025). Therefore, the lack of a summer harvesting effect on winter deaths may be due to a persistent or renewed pool of season-specific vulnerabilities, rather than a simple depletion of frail individuals. These findings align with earlier studies showing that heat has a short-term impact, while cold exerts a more prolonged effect (Ferreira Braga et al., 2001).

Studying the harvesting effect is crucial for understanding the relationship between climate change and demographic behaviour. It sheds light on the phenomenon where external stressors, such as extreme weather events, heatwaves, or other health crises, accelerate deaths among individuals who are already vulnerable or near the end of their lifespan (Ha et al., 2011; Rocklöv et al., 2009; Stafoggia et al., 2006). Exploring the harvesting effect enables researchers to accurately assess the immediate and long-term implications of such events on population dynamics and health outcomes (Toulemon & Barbieri, 2008). This knowledge can inform targeted public health strategies, including strengthening early warning systems, improving access to cooling and heating for older adults, enhancing surveillance of temperature-related mortality, and improving communication campaigns to the most vulnerable subgroups. These findings also highlight the need to integrate climate-related mortality risks into Italy’s regional adaptation plans and health system planning.

The limitations of the current study must also be acknowledged. The monthly mortality data by province was available for only eight complete winters, from 2011 to 2019. If there is an adaptation to extreme temperature-related mortality, this is unlikely to be detected in such a short time interval observed in our study. Furthermore, the cause-specific mortality data were not available for all provinces during the study period. Having access to such data could have provided greater insight on cardiovascular and respiratory mortality, which are known from the existing literature to exhibit the highest seasonality in mortality. We use provinces as the geographical unit because province-level mortality data by age and sex are the most granular and nationally consistent data available for all of Italy during 2011–2019. Although some city-specific studies employ finer spatial units, these data are not available uniformly across the country.

Despite its limitations, this study offers important insights into assumptions about future mortality risks under climate change. While we find some evidence of a harvesting effect where high winter mortality reduces the pool of vulnerable individuals, we also observe that mortality risks remain elevated during extremely hot days (above the 95th percentile). As Italy is expected to experience not only more frequent but also more intense heatwaves in the coming decades (Sabelli, 2023), this suggests that the harvesting effect may no longer mitigate mortality following severe winter events. Instead, the rising intensity of heat exposure will likely lead to higher mortality, affecting a broader segment of the population. This finding indeed carries significant implications for population dynamics. If climate change increases mortality risks, then population projections such as those by the United Nations, the Wittgenstein Centre for Demography and Global Human Capital, and national statistical offices should incorporate climate-related mortality effects into their models.

In this paper, we present evidence that extreme temperatures, both cold and hot, continue to pose serious mortality risks in Italy. Importantly, we show that even under the current level of global warming, the harvesting effect does not offset the mortality associated with extreme heat. Looking ahead, in a future climate characterised by more frequent and intense heat events, it becomes crucial to develop strategies to reduce their impact on mortality. In fact, most heat-related deaths are preventable through timely interventions and effective warning systems. However, implementation remains challenging due to the short time window between exposure and death. Prevention efforts must therefore focus on the most vulnerable populations and be based on a clear understanding of high-risk conditions (Basu & Samet, 2002). Surveillance systems are essential components of prevention plans as they can provide valid data to quickly assess the health impact of extreme weather event. Temperature-related mortality remains a pressing public health concern and should and should be treated as such by policymakers and health authorities. This research highlights the urgent need for adaptive measures and targeted public health interventions to protect vulnerable population and reduce the health burden of extreme temperatures in Italy.

## Data Availability

https://www.istat.it/en/news/mortality-data/ Mortality and population data at the provincial level were obtained from ISTAT in the related websites https://www.istat.it/notizia/dati-di-mortalita-cosa-produce-listat/ (mortality) and https://demo.istat.it/ (population). Meteorological data (temperature, precipitation, humidity, solar radiation, and wind speed) were retrieved from the E-OBS dataset via the Copernicus Climate Data Store: https://cds.climate.copernicus.eu/. PM₂.₅ data were sourced from the CAMS global reanalysis via the Copernicus Atmosphere Data Store: https://ads.atmosphere.copernicus.eu/

## Appendix

See Tables 2, 3, 4, 5, 6, 7, 8, 9, 10, 11 and Fig. 6.

**Table 2:** Extreme temperature and mortality.

	(1)
< 5th percentile	0.002***
	(0.001)
5th to 10th percentile	0.001
	(0.001)
10th to 15th percentile	0.001
	(0.001)
15th to 20th percentile	0.001
	(0.001)
80th to 85th percentile	− 0.000
	(0.000)
85th to 90th percentile	0.001***
	(0.000)
90th to 95th percentile	0.001
	(0.001)
> 95th percentile	0.005***
	(0.001)
Solar radiation	0.000***
	(0.000)
Precipitation	0.000
	(0.001)
Wind speed	− 0.003
	(0.004)
Humidity	0.001***
	(0.000)
PM2.5	0.001***
	(0.001)
70–79	1.009***
	(0.003)
80–89	2.230***
	(0.006)
90+	3.365***
	(0.010)
Female	− 0.420***
	(0.004)
Observations	79,608

**Table 3:** Extreme temperature and mortality in the summer season and interaction with the previous high winter mortality.

	(1)
80th to 85th percentile	− 0.001
	(0.001)
80th to 85th percentile × high winter mortality	0.001
	(0.001)
85th to 90th percentile	0.001
	(0.001)
85th to 90th percentile × high winter mortality	− 0.001
	(0.001)
90th to 95th percentile	0.002***
	(0.001)
90th to 90th percentile × high winter mortality	− 0.002***
	(0.001)
> 95th percentile	0.004***
	(0.001)
> 95th percentile × high winter mortality	0.000
	(0.001)
Observations	20,544

**Table 4:** Extreme temperature and mortality in the summer season and interaction with the previous high winter mortality by age.

	(1)	(2)	(3)	(4)
	60–69	70–79	80–89	90+
80th to 85th percentile	− 0.001	− 0.001	− 0.002	0.001
	(0.002)	(0.002)	(0.001)	(0.002)
80th to 85th percentile × high winter mortality	− 0.003	− 0.000	0.003	− 0.000
	(0.004)	(0.003)	(0.002)	(0.002)
85th to 90th percentile	0.000	0.000	0.000	0.004***
	(0.002)	(0.002)	(0.001)	(0.001)
85th to 90th percentile × high winter mortality	0.001	− 0.000	0.000	− 0.004***
	(0.003)	(0.002)	(0.002)	(0.002)
90th to 95th percentile	0.002	0.000	0.003***	0.003***
	(0.002)	(0.002)	(0.001)	(0.001)
85th to 90th percentile × high winter mortality	− 0.006***	− 0.000	− 0.002	− 0.003
	(0.002)	(0.002)	(0.001)	(0.001)
> 95th percentile	− 0.000	0.005***	0.004***	0.006***
	(0.002)	(0.002)	(0.001)	(0.002)
> 95th percentile × high winter mortality	− 0.000	− 0.002	0.002	0.000
	(0.002)	(0.001)	(0.001)	(0.001)
Solar radiation	0.000	0.000	0.000***	− 0.000
	(0.000)	(0.000)	(0.000)	(0.000)
Precipitation	0.005	− 0.001	0.003	− 0.005
	(0.005)	(0.003)	(0.002)	(0.004)
Wind speed	0.010	− 0.001	− 0.008	− 0.004
	(0.016)	(0.011)	(0.009)	(0.010)
Humidity	0.000	0.003***	0.000	− 0.000
	(0.001)	(0.001)	(0.001)	(0.001)
PM2.5	0.002	0.002	0.006***	0.009***
	(0.002)	(0.002)	(0.001)	(0.002)
Female	− 0.623***	− 0.560***	− 0.334***	− 0.172***
	(0.010)	(0.007)	(0.006)	(0.006)
Observations	5,136	5,136	5,136	5,136

**Table 5:** Extreme temperature and mortality in the winter season and interaction with the previous summer season.

	(1)
< 5th percentile	0.002***
	(0.001)
< 5th percentile × high summer mortality	− 0.001
	(0.001)
5th to 10th percentile	− 0.000
	(0.001)
5th to 10th percentile × high summer mortality	0.000
	(0.001)
10th to 15th percentile	0.001
	(0.001)
10th to 15th percentile × high summer mortality	− 0.001
	(0.001)
15th to 20th percentile	0.002
	(0.001)
15th to 20th percentile × high summer mortality	− 0.002
	(0.001)
Observations	20,544

**Table 6:** Effect modification of previous winter mortality on the impact of PM2.5 on mortality, including an interaction term between winter mortality and PM2.5.

	(1)
80th to 85th percentile	− 0.000
	(0.001)
80th to 85th percentile × high winter mortality	0.000
	(0.001)
85th to 90th percentile	0.002***
	(0.001)
85th to 90th percentile × high winter mortality	− 0.001
	(0.001)
90th to 95th percentile	0.003***
	(0.001)
85th to 90th percentile × high winter mortality	− 0.002***
	(0.001)
> 95th percentile	0.005***
	(0.001)
> 95th percentile × high winter mortality	0.000
	(0.001)
PM2.5	0.005***
	(0.001)
PM2.5 × high winter mortality	− 0.002***
	(0.001)
Observations	20,544

**Table 7:** Extreme temperature and mortality and interaction with age.

	(1)
< 5th percentile	− 0.003***
	(0.001)
< 5th percentile × age 70–79	0.002***
	(0.001)
< 5th percentile × age 80–89	0.006***
	(0.001)
< 5th percentile × age 90+	0.009***
	(0.001)
5th to 10th percentile	0.001
	(0.001)
5th to 10th percentile × age 70–79	− 0.001
	(0.001)
5th to 10th percentile × age 80–89	− 0.000
	(0.001)
5th to 10th percentile × age 90+	0.001
	(0.001)
15th to 20th percentile	− 0.003***
	(0.001)
10th to 15th percentile × age 70–79	0.004***
	(0.001)
10th to 15th percentile × age 80–89	0.004***
	(0.001)
10th to 15th percentile × age 90+	0.004***
	(0.001)
15th to 20th percentile	− 0.005***
	(0.001)
15th to 20th percentile × age 70–79	0.002
	(0.001)
15th to 20th percentile × age 80–89	0.007***
	(0.001)
15th to 20th percentile × age 90+	0.011***
	(0.001)
80th to 85th percentile	0.003***
	(0.001)
80th to 85th percentile × age 70–79	− 0.001
	(0.001)
80th to 85th percentile × age 80–89	− 0.003***
	(0.001)
80th to 85th percentile × age 90+	− 0.004***
	(0.001)
85th to 90th percentile	0.003***
	(0.001)
85th to 90th percentile × age 70–79	− 0.002
	(0.001)
85th to 90th percentile × age 80–89	− 0.003***
	(0.001)
85th to 90th percentile × age 90+	− 0.002
	(0.001)
90th to 95th percentile	0.001
	(0.001)
90th to 95th percentile × age 70–79	0.001
	(0.001)
90th to 95th percentile × age 80–89	0.001
	(0.001)
90th to 95th percentile × age 90+	− 0.001
	(0.001)
> 95th percentile*	0.001
	(0.001)
> 95th percentile × age 70–79	0.002***
	(0.001)
> 95th percentile × age 80–89	0.004***
	(0.001)
> 95th percentile × age 90+	0.007***
	(0.001)
Observations	79,608

**Table 8:** Extreme temperature and mortality and interaction with sex.

	(1)
< 5th percentile	0.001
	(0.001)
< 5th percentile × female	0.002***
	(0.000)
5th to 10th percentile	0.000
	(0.001)
5th to 10th percentile × female	0.000
	(0.001)
10th to 15th percentile	0.000
	(0.001)
10th to 15th percentile × female	0.001
	(0.001)
15th to 20th percentile*	− 0.000
	(0.001)
15th to 20th percentile × female	0.003***
	(0.001)
80th to 85th percentile	− 0.000
	(0.001)
80th to 85th percentilev	− 0.000
	(0.001)
85th to 90th percentile	0.001
	(0.001)
85th to 90th percentile × female	0.001
	(0.001)
90th to 95th percentile	0.002***
	(0.001)
90th to 95th percentile × female	− 0.001
	(0.001)
> 95th percentile	0.003***
	(0.001)
> 95th percentile × female	0.004***
	(0.000)
Observations	79,608

**Table 9:** Extreme temperature and mortality and interaction with sex.

	(1)
< 5th percentile	− 0.000
	(0.001)
< 5th percentile × hot	0.003***
	(0.001)
< 5th percentile × temperate	0.003***
	(0.001)
5th to 10th percentile	0.001
	(0.001)
5th to 10th percentile × hot	− 0.000
	(0.001)
5th to 10th percentile × temperate	− 0.001
	(0.001)
10th to 15th percentile	0.000
	(0.001)
10th to 15th percentile × hot	0.002
	(0.001)
10th to 15th percentile × temperate	0.000
	(0.001)
15th to 20th percentile	0.001
	(0.001)
15th to 20th percentile × hot	0.001
	(0.001)
15th to 20th percentile × temperate	− 0.000
	(0.001)
80th to 85th percentile	0.000
	(0.001)
80th to 85th percentile × hot	− 0.000
	(0.001)
80th to 85th percentile × temperate	− 0.000
	(0.001)
85th to 90th percentile	0.001
	(0.001)
85th to 90th percentile × hot	0.002
	(0.001)
85th to 90th percentile × temperate	0.000
	(0.001)
90th to 95th percentile	0.000
	(0.001)
90th to 95th percentile × hot	0.002
	(0.001)
90th to 95th percentile × temperate	0.001
	(0.001)
> 95th percentile	0.005***
	(0.001)
> 95th percentile × hot	0.001
	(0.001)
> 95th percentile × temperate	− 0.000
	(0.001)
Observations	79,608

**Table 10:** Extreme temperature, interaction with winter mortality by Climatic zones.

	(1)	(2)	(3)
	Cold	Hot	Temperate
80th to 85th percentile	− 0.000	− 0.001	− 0.002
	(0.002)	(0.001)	(0.001)
80th to 85th percentile × high winter mortality	− 0.003	0.001	0.001
	(0.004)	(0.002)	(0.002)
85th to 90th percentile	− 0.001	0.001	0.000
	(0.002)	(0.002)	(0.001)
85th to 90th percentile × high winter mortality	− 0.000	− 0.002	− 0.001
	(0.003)	(0.002)	(0.001)
90th to 95th percentile	0.003	0.002	0.002
	(0.003)	(0.001)	(0.001)
90th to 90th percentile × high winter mortality	− 0.002	− 0.001	− 0.003
	(0.002)	(0.001)	(0.002)
> 95th percentile	0.005	0.003***	0.002
	(0.003)	(0.001)	(0.002)
> 95th percentile × high winter mortality	− 0.001	− 0.000	0.001
	(0.003)	(0.001)	(0.001)
Observations	20,544		

**Table 11:** 5th and 95th percentiles of mean temperature in Italian provinces.

Province	5th percentile (°C)	95th percentile (°C)
Agrigento	8.21	26.84
Alessandria	2.53	24.97
Ancona	3.73	26.32
Arezzo	1.62	23.96
Ascoli Piceno	2.81	25.28
Asti	3.03	25.07
Avellino	3.99	24.54
Bari	6.7	26.68
Barletta-Andria-Trani	6.85	27.06
Belluno	− 4.43	18.54
Benevento	4.56	25.34
Bergamo	− 0.45	22.06
Biella	0.57	22.69
Bologna	1.66	26.03
Bolzano-Bozen	− 6.99	15.62
Brescia	− 0.61	22.67
Brindisi	8.07	27.28
Cagliari	7.71	26.94
Caltanissetta	8.08	26.82
Campobasso	4.31	25.75
Caserta	5.99	26.08
Catania	7.01	25.83
Catanzaro	5.8	25.24
Chieti	4.05	25.56
Como	0.73	22.76
Cosenza	4.39	24.66
Cremona	1.17	26.7
Crotone	7.24	27.14
Cuneo	0.62	21.24
Enna	6.85	25.6
Fermo	3.91	26.43
Ferrara	1.76	26.53
Firenze	1.99	24.16
Foggia	6.12	26.84
Forlì-Cesena	1.89	25.28
Frosinone	4	25.27
Genova	1.77	22.79
Gorizia	1.8	25.47
Grosseto	4.64	25.03
Imperia	4.1	23.21
Isernia	2.89	24.2
L’Aquila	0.5	22.03
La Spezia	1.6	23.39
Latina	6.38	25.86
Lecce	8.15	27.85
Lecco	− 0.05	22.07
Livorno	5.39	25.46
Lodi	1.38	26.99
Lucca	1.28	23.16
Macerata	2.38	25.34
Mantova	1.25	26.9
Massa-Carrara	0.58	22.84
Matera	6.7	26.88
Messina	6.09	24.79
Milano	1.77	26.58
Modena	1.05	25.25
Monza e della Brianza	1.82	26.21
Napoli	6.88	26.4
Novara	1.59	25.22
Nuoro	5.87	25.04
Oristano	7.38	26.37
Padova	1.63	26.44
Palermo	6.71	25.46
Parma	0.98	24.53
Pavia	1.76	26
Perugia	2.22	24.64
Pesaro e Urbino	3.03	25.68
Pescara	3.62	25.41
Piacenza	1.13	24.8
Pisa	4.06	25.19
Pistoia	1.44	23.12
Pordenone	− − 0.73	23.08
Potenza	3.84	24.16
Prato	1.84	24.04
Ragusa	8.49	27.37
Ravenna	2.21	26.33
Reggio Calabria	6.1	25.26
Reggio nell’Emilia	1.21	25.37
Rieti	1.56	22.89
Rimini	2.85	26.03
Roma	5.44	25.99
Rovigo	1.82	26.66
Salerno	5.16	24.65
Sassari	7.05	25.56
Savona	3.72	23.73
Siena	3	24.61
Siracusa	8.62	27.55
Sondrio	− 6.81	14.96
Sud Sardegna	7.32	26.65
Taranto	7.62	27.27
Teramo	2.79	24.95
Terni	3.22	25.19
Torino	− 1.15	20.44
Trapani	8.81	27.25
Trento	− 3.97	18.4
Treviso	1.12	25.31
Trieste	1.44	24.39
Udine	− 1.63	21.85
Valle d’Aosta	− 7.41	13.98
Varese	1.82	24.62
Venezia	1.85	26.16
Verbano-Cusio-Ossola	− 3.22	18.24
Vercelli	0.54	22.79
Verona	1.4	25.69
Vibo Valentia	5.83	24.64
Vicenza	0.46	23.98
Viterbo	4.66	25.44

## Footnotes

1 In this article, “sex” refers to the biological characteristics that define humans as male or female, including chromosomal, hormonal, and anatomical differences.

## Material Availability

https://cds.climate.copernicus.eu/ All raw datasets are publicly accessible.

## Code Availability

All analysis code is available in our OSF repository: https://osf.io/xjda8
